# Risk communication during disease outbreak response in post-Ebola Liberia: experiences in Sinoe and Grand Kru counties

**DOI:** 10.11604/pamj.supp.2019.33.2.16877

**Published:** 2019-05-28

**Authors:** John Sumo, Geraldine George, Vera Weah, Laura Skrip, Julius Monday Rude, Peter Clement, Jeremias Domingos Naiene, Liliane Luwaga, Joseph Chukwudi Okeibunor, Ambrose Talisuna, Ali Ahmed Yahaya, Soatiana Rajatonirina, Musoka Fallah, Tolbert Nyenswah, Bernice Dahn, Alex Gasasira, Ibrahima Socé Fall

**Affiliations:** 1 Health Promotion Division, Ministry of Health, Monrovia, Liberia; 2 National Public Health Institute, Monrovia, Liberia; 3 World Health Organization, Monrovia, Liberia; 4 World Health Organization, Regional Office for Africa, Brazzaville, Congo; 5 Ministry of Health, Monrovia, Liberia

**Keywords:** Risk communication, Liberia, outbreak, Sinoe, Grand Kru, interpersonal communication (IPC)

## Abstract

**Introduction:**

Lessons learned from the Ebola virus disease (EVD) outbreak enabled Liberia to develop a health plan for strengthening public health capacity against potential public health threats. risk communication is one of the core pillars that provide life-saving information and knowledge for the public to take preventive and proactive actions against public health threats. These were applied in response to the post-ebola meningococcal septicemia and meningitis outbreaks in Sinoe and Grand Kru counties. This paper documents risk communication experiences in these post-ebola outbreaks in Liberia.

**Methods:**

Risk Communication and health promotion strategies were deployed in developing response plans and promptly disseminating key messages to affected communities to mitigate the risks. Other strategies included engagement of community leaders, partnership with the media and dissemination of messages through the community radios, active monitoring community risk perceptions and compliance, rumor management, mobile stage and interpersonal communication (IPC) during the Meningococcal disease outbreaks in Sinoe and Grand Kru counties.

**Results:**

In Sinoe, about 36,891 households or families in 10 health districts were reached through IPC and dialogue. Circulating rumors such as “Ebola” was the cause of deaths was timely and promptly mitigated. There was increased trust and adherence to health advice including prompt reporting of sick people to the nearest health facility in the two counties.

**Conclusion:**

Risk communication and health promotion encouraged community support and involvement in any response to public threats and events. No doubt, risk communication and health promotion play an important role in preparedness and response to public health emergencies.

## Introduction

The magnitude and intensity of the 2014-2016 Ebola virus disease (EVD) outbreak in Liberia incapacitated the country’s social, educational, and financial sectors. The health system was overwhelmed by the high case counts, inadequate isolation capacity, and staggering death toll among infected healthcare workers. By the end of the outbreak, a total of 9,862 confirmed, suspected, and probable EVD cases with 4,408 deaths had been registered [1]. The health system was ill-prepared, partly due to limited resources and weaknesses in the core competencies of the International Health Regulations (IHR) namely the ability to detect, prevent and respond to public health threats. Like most sectors within the health system, the 2014 outbreak of the ebola virus uncovered the multiple challenges and weaknesses within Liberia’s Health Promotion Division (NHPD). As a result, initial efforts of the NHPD to respond to concerns of the general population were overwhelmed by rumors and distrust. Contributing to the rapid spread of transmission, the initial response to the EVD outbreak was characterized by high levels of public denial, misconceptions, misinformation and resistance at the community level. Findings from anthropological studies conducted during the outbreak provided additional insight into community perspectives of the disease and the response efforts [2, 3]. Many community members attributed their initial disbelief of ebola either to the clinical presentation of the disease, which had symptoms similar to common illnesses like malaria and cholera, or to supernatural causes. Additionally, several conspiracy theories were circulating and suggested that Ebola was a manmade disease or money-making scheme. Moreover, community members complained that government messages about Ebola were ambiguous and confusing e.g. calling for the sick people to go for the health facilities and yet the messages were informing the community that “Ebola kills and has no cure”. While the media initially focused on debating on misuse of resources as opposed to promoting key messages diverting the response efforts, there was also poor coordination among partners which resulted in the dissemination of conflicting messages. Although community concerns were not captured to inform the messages and approaches initially, this was adjusted using results from the anthropological studies [3].

Wildly spreading rumors and misinformation about the virus also adversely affected response efforts. Some communities were reluctant to report sick loved ones and relatives for fear of being quarantined or taken to the Ebola treatment unit (ETUs). More deaths occurred at homes and secret burials were frequently practiced to avoid the government’s cremation policy [2]. Hence, interrupting the transmission of the virus became complex. It became imperative to introduce risk communication as a sure way of providing real-time information and easily understood messages to the public about Ebola and of encouraging participation in the response efforts. In October 2014, Liberia’s NHPD initiated its Ebola-related risk communication activities. Messaging prompted people to observe preventive measures including washing of hands with soap and water, not touching sick ones suspected of having the disease, allowing decent and safe burials, identifying sick people within the community, and calling the EVD hotline to pick up sick people from the community. Despite progress, the health promotion efforts continued to encounter misconceptions, doubts, and community resistance before Liberia was declared Ebola-free in May 2015 [4]. Three subsequent EVD outbreaks in Margibi and Montserrado counties during 2015 and 2016 provided the NHPD with additional opportunities to improve its health promotion response strategies.

Based on the lessons learned from the EVD outbreak in the West African Region, WHO/AFRO organized a three-day meeting in 2015 in Dakar, Senegal with the ultimate goal of strengthening national Risk Communication under the 2005 International Health Regulations (IHR) in each Ebola-affected country. Risk Communication stands out as a core element of the IHR to detect, report, and respond to any public health emergency [5]. WHO and CDC supported Liberia’s capacity building and strengthening. WHO supported 55 master trainers as trainers of trainers (TOTs) from the National Health Promotion Division, County Health Promotion Focal persons and program managers; 75 media personnel, and 19 community health and health promotion focal persons from the counties. Additionally, in April 2017, the CDC trained 53 health promotion and community health focal persons and other central level staff in field module risk Communication [6-8] With this capacity, the NHPD was better positioned to respond swiftly during outbreaks. The health promotion focal persons from Sinoe, Grand Kru and other Counties were trained in social mobilization during the EVD outbreaks and gain a lot of experiences in providing messages to reduce fear and convince the communities to visit the health facilities [9]. However, the need of a risk communication strategies to address similar outbreaks in future was one of the lessons learnt during the EVD outbreak [10-16]. The post-EVD outbreaks in Sinoe and Grand Kru Counties were promptly addressed using the risk communication strategies, which was useful also to reduce the fear and enhance EVD surveillance during the flare ups in other counties [17]. However, there is a limited documentation of the levels of effectiveness of the risk communication strategies to provide lessons to other public health interventions within and outside Liberia. This paper thus documents the role of risk communication in response efforts during post-EVD outbreaks of meningococcal septicemia in Sinoe and meningitis in Grand Kru Counties.

## Methods

Under the IHR, risk communication for public health emergencies includes the range of communication capacities required through the preparedness, response and recovery phases of a public health event to encourage informed decision-making, positive behavior change and the maintenance of trust. Risk communication outbreak responses for Sinoe and Grand Kru were mainly characterized by the practical application of lessons learnt from the Ebola outbreak with additional skills from the trainings conducted. The main risk communication methods used in Sinoe County Meningococcal Septicemia and the Grand Kru Meningitis outbreaks were centered around active community engagement of affected and surrounding communities through the development of messages, advocacy with local stakeholders, interpersonal communication , live radio talk shows in English and local languages (Kru and Sapo), and active monitoring and response to rumors. All responses were to promptly address community concerns, and link messages and actions to the desired practices that reduce the risk of transmission within the community. Key messages and materials informed by the risk assessment and community risk perceptions were developed and disseminated in the affected counties through appropriate channels to enable community members to identify risky practices and reduce the risks of transmission and spread of the disease within the community. Additionally, targeted community engagement strategy with key stakeholders including county authorities, chiefs and elders, and community leaders was useful to change community perceptions and myths and for them to solicit their support and participation in response efforts in the two counties. Regular updating of the coordination and community engagement pillars on community concerns and potential barriers to compliance and control measurers helped tailor the response strategies.

### Sinoe county outbreak of meningococcal septicemia

**Description of outbreak:** on tuesday, 25th April 2017, the Sinoe County Health Team (SCHT) notified the National Public Health Institute of Liberia (NPHIL) and the Ministry of Health (MoH) of a cluster of unexplained health events involving 14 cases with 9 deaths in Greenville City. According to the SCHT, cases presented with headache, diarrhea, mental confusion, weakness, vomiting and abdominal pain. Many of the cases reported onset of symptoms following attendance at a funeral event (wake, burial, repass) on the 21st and 22nd of April 2017. The disease was initially characterized by high case fatality and the need to disseminate information despite the uncertainty of the disease’s causative agent (Table 1). Due to the magnitude of the outbreak, the NPHIL and the MoH deployed surge capacity with a team of six national experts providing technical support to the SCHT. The team establishment was based on call for national support in the areas of coordination, infection prevention and control, case management, dead body management, social mobilization/risk communication, and epidemiology. In view of the convolution of the outbreak, the national and county Incident Management Systems (IMS) were activated along with the district rapid response team (RRT) backed by the national technical support team. Needed resources including medical and non-medical supplies were mobilized. Strategic areas of focus for the investigation included social investigations (social epidemiology) and disease investigations (disease epidemiology including laboratory). The initial investigation sites included eight communities in the Greenville health district: Teah´s Town, Red Hill, Congo Town, Down Town, Johnston Street, Mississippi Street, PO-River, and Fish Town communities. Other communities visited during the investigation included Louisiana, Butwua Oil Plantation Company (BOPC) mining/plantation, and Dioh Town. Using the ring approach, the response team was able to identify all facilities used for treatment including public and private health facilities, traditional healing places and healers, and prayer homes. All neighboring counties were alerted and regular updates shared.

**Table 1:** Chronology of the main events and activities implemented during the meningococcal disease outbreak, Sinoe County, Liberia, 2017

Date	Events and activities
25 April 2017	Sinoe County Health Team (SCHT) notified the National Public Health Institute of Liberia (NPHIL) and the Ministry of Health (MoH) of a cluster of unexplained health events involving 14 cases with 9 deaths in Greenville City
25 April 2017	Activation of the incident management system at the County level
25 April – 23 June 2017	Community Engagement meetings with Religious leaders, Chiefs and Elders, Schools and youth groups
26 April - 26 June 2017	House to house awareness by mobilizers
26 April – 26 June 2017	Publicity awareness with PA system in moving Vehicle
29 April 2017	3 suspected cases self-reported to the main hospital (FJ Grante hospital) in Sinoe County
30 April 2017	Last case of the outbreak reported
3 May – 25 June 2017	Radio Talk shows
3 May 2017- 25 June 2017	Radio announcement with jingles
7 May 2017	Outbreak confirmed as meningoccocal disease
14 May 2017	County Level Advocacy Meeting
22 - 25 May 2017	District Advocacy Meetings
25 May 2017	Provision of chemoprofilaxis with ciprofloxacin and ceftraxone to the contacts without any community resistance reported

### Grand Kru county outbreak of meningitis

**Description of outbreak:** on September 22, 2017, the Grand Kru County Health Team (GKCHT) reported three suspected cases of meningitis that were detected in Barclayville Health District. The index case was a 22-year-old female client who was admitted on September 7, 2017 but died September 9, 2017, two days after admission in the hospital. Burial was conducted by the patient’s family member who did not observe the safe and dignified burial procedure. Meanwhile no other family member reported similar symptoms. On September 17, 2017, an 18-year-old male was confirmed of meningitis in the Zoloken community in Barclayville Health District, Grand Kru. In all, there were five other cases including one confirmed, 3 suspected and one death recorded. The cases presented with common signs and symptoms like headache, stiff neck, altered level consciousness, fever, inability to talk, etc. Blood samples were collected for all three of the cases and transported to the Nation Reference Laboratory in Margibi County. However, only one case was confirmed meningitis positive. Fifty contacts were line-listed including 17 health workers.

## Results

**Mechanisms of risk communication:** during the outbreak intervention, the County IMS was activated to coordinate the response activities. Various risk communication approaches were deployed to respond to the cluster of deaths in the county. Risk assessment, understanding community risk perceptions, advocacy meetings, targeted community engagements with influencers, inter-personal communication, and media engagements were conducted to seek communities’ support and participation in the response. The risk communication team engaged community and religious leaders to create awareness, dispel rumors and overcome community resistance. Information was provided to the public through radio talk shows and street broadcasters, encouraging the community to identify and report persons with specific signs and symptoms in the community to health facilities. A town hall meeting was convened to solicit information from community members and for the county superintendent and health officials to answer questions from the public. Properly trained social mobilizers worked jointly with surveillance officers during active case search, facilitating entry and surveillance activities in the community. Consequently, community leaders and members received real time information on the cluster of deaths and they supported preventive measures to address the health problem. Sinoe County has two major radio stations situated in the capital Greenville: the Liberia Broadcasting System (LBS) and the Sinoe County Community Radio Station (SCCRS). These stations cover all of the ten health districts. However, both radio stations had technical difficulties during the first week of the response. The mobile stage and the SCCRS were instrumental in disseminating the meningitis prevention messages. The mobile stage consisted of a pick-up truck on which loud speakers were mounted. The truck moved in and around Greenville City and aired key messages on hand washing, the importance of reporting anyone showing signs and symptoms to community leaders or health authorities, and prevention messages on meningitis. This was reinforced by radio talk shows that included discussions of these messages. Local community radio stations also aired similar messages as public service announcements. The LBS, the mobile stage and the SCHT’s daily Situational Report were the basic media outlets used in the response, especially in providing real time information and addressing rumours.

**Content of risk communication messages:** key messages were tailored to the stage of the response. These included emphasizing importance of good hygiene practices such as hand washing, avoiding shaking of hands and public gatherings, and the need to report to a health facility upon onset of any symptoms. Due to the uncertainty of the cause of death, the initial risk communication messages focused on reassuring the public that the blood samples tested negative for Ebola. The main content of the messages was for communities to report any usual deaths to the health facilities. Due to the breakdown of the only functional radio station in the county during first week of the response, a mobile stage was used to broadcast the message and the negative Ebola laboratory results to the 10 hotspots and 14 surrounding communities in Greenville and in the Butwua and Kpan Districts. Moreover, materials such as hand washing posters and brochures were used during community engagement meetings.

**Staffing:** risk communication response activities were led by the county health promotion focal person. Additional technical support was provided by the NHPD, WHO, CDC and UNICEF. The Risk Communication Focal Person was on the ground working with the Sinoe county social mobilization pillar. The SCHT with support from the MOH at the central level and UNICEF deployed community health volunteers who conducted house-to-house awareness in districts and at the community level. These activities were coordinated by the county health promotion focal person and the community health focal persons.

**Outcomes of the risk communication strategy**: through the joint efforts of all thematic response pillars, county authorities and the national rapid response team, a total of 27 cases with 10 deaths were reported in Sinoe County and four epidemiologically-linked cases, including three deaths, were reported in Montserrado and Grand Bassa counties. Cases were managed, and the outbreak contained only among those who attended the funeral. Rumors that Ebola was the cause the death that circulated were timely and promptly mitigated. About 36,891 households or families were reached through interpersonal communication; this led to increased trust and adherence including prompt reporting of sick people to the nearest health facility (Table 2).

**Table 2:** Main outcomes of the risk communication and social mobilization activities implemented during the meningococcal disease outbreak, Sinoe County, Liberia, 2017

Unit	Total Number
Estimated household/families reached through IPC	36891
Estimated children reached through house to house mobilization	15229
Estimated Women reached through house to house visits	19323
Total community meetings held	821
Estimated homes hand washing stations reactivated	36891

**Description of risk communication strategy**: similar to the Sinoe outbreak, the GKCHT activated the County IMS and key response pillars: coordination, surveillance, risk communication/ social mobilization, and infection prevention and control. The risk communication plan was produced and implemented quickly to develop and disseminate key messages on meningitis in all the districts through engagement meetings with key stakeholders in communities and schools. Meningitis messages including the factsheets and audios messages about the disease were disseminated to the county in less than 24 hours. The Voice of Grand Kru, a local community radio station in Grand Kru supported by UNICEF was used to air messages. The county health promotion focal person led the risk communication team along with community health focal person. Other channels used to share information were through community engagement meetings, interpersonal communication by community health assistants and local leaders.

Content of risk communication messages: using lessons learned from previous outbreaks including the meningococcal septicemia outbreak in Sinoe, stakeholders’ advocacy, community engagement meetings in schools and town halls, radio talk shows, airing of jingles were the medium through which messages were disseminated during the Grand Kru Meningitis cases. Message content informed the at-risk population about the disease, the signs and symptoms, and importance of early reporting to the nearest health facilities.

**Staffing:** prior to the outbreak, meningitis messages were developed by the national a message coordination body at the national level. Through the chair of the Social Mobilization/Risk Communication Pillar, messages were promptly disseminated from the national level to the county. Messages were promptly disseminated because of the timely sharing of the laboratory results and availability of the messages prior to the cases confirmation. The County risk Communication Pillar team which was led by the Health Promotion Focal Person along with the Community Health Focal Person oversaw the communication aspect of the response. Community Health Assistants were responsible for house-to-house awareness. The voice of Grand Kru, the local radio station, played a key role in disseminating the meningitis messages. Live radio talk shows were conducted by member of the county health team and stakeholders.

**Outcomes of risk communication strategy:** as the result of the robust approach and coordination between major response pillars and with the national and county coordination mechanism, the outbreak was timely interrupted. As of the first three cases reported, no additional cases were detected among 50 line-listed contacts. The community members in the three communities were well-informed about the disease and took preventive measures. The voice of Grand Kru was the main source of information sharing during the response activities. The total summary of the population reached remained a major challenge due to a gap in national monitoring. At least 50 contacts including 17 health care workers from the Sass Town Health Center and Rally Town Hospital were line- listed. Of the total 5 cases suspected, only 1 was confirmed meningitis positive. There was only one death and no secondary transmission was reported among cases. Risk communication played a significant role during and after the response. Community members and elders helped in identifying people connected with the health problems and persons infected or suspected of being infected sought medical attention at various health centers in the county. Indeed “local solutions helped solve local problems!” Risk communication increased the level of trust in the health system contrary to the perceptions exhibited during the EVD outbreak.

## Discussion

Overall, risk communication has played a key role in post-EVD recovery in Liberia. Successful strategies that were deployed during the EVD response in West Africa [16, 18] have been repeatedly applied to non-EVD outbreak settings. As was seen towards the end of the initial EVD outbreak in Liberia and again during subsequent flare-ups, key meningitis prevention messages and materials were developed and disseminated during and after the outbreaks in Sinoe and Grand Kru Counties. Community members who were exposed to these messages got a clear understanding of the disease that affected them. Other risk communication milestones included community dialogues and interpersonal communication with key stakeholders including at-risk segments of communities where issues were discussed and consensus reached on key action plan/ practices to reduce the risk. Community members actively assisted in identifying suspected cases and reporting them to health authorities while they reactivated home hand washing stations. The dissemination of the meningitis prevention messages through different channels like radio health talks shows and the mobile stage also buttressed the response efforts in the two counties. This helped to quickly address rumors and misinformation (Sinoe and Grand Kru Health teams personal communication). The risk communication strategies were effective to control other outbreaks during the late stage and after EVD outbreak in other counties of Liberia as well, with example of Lassa fever outbreak in 2016 when the communities had lack of knowledge of the disease in the beginning of the outbreak [19].

The outbreaks in Sinoe and Grand Kru Counties provided important lessons regarding the factors that can either hinder or motivate community members’ willingness to adhere to risk communication messages during an outbreak. In Sinoe, information regarding the cause of the health emergency was initially confusing and, in some cases, contradictory. Initially, the cause of the unexplained illnesses and cluster deaths were reported as “probable meningitis”. The public was subsequently informed that the cause of the deaths and illnesses was meningitis. Consequently, the public was confused and skeptical about the prevailing health problem until it was officially confirmed as meningococcal septicemia and messages clarified thereafter [20]. Best practices observed during the response involved community leaders and health workers collaboratively addressing problems. Community leaders and key stakeholders all worked together as a team with one goal in mind: to mitigate the health problems that affected them. Prompt response with key messages helped to respond to the problems. In this context, risk communication using appropriate messages, and the airing of same, community engagement and addressing community concerns, rumors and misinformation should form part of future emergency response. There were several limitations to the outbreak response that are important to mention. The dissemination of information about the outbreak in Sinoe County took some time and even with the information people did not and still do not know the actual cause of the reported meningococcal septicemia. Hence, in Sinoe County, it was difficult to convey the appropriate message to the public. However, in Grand Kru, where the initial case was a confirmed case of meningitis, the information about the disease and how to prevent additional cases was clear from the outset.

Lessons learned: early confirmation of a disease outbreak helps to provide a quick and coordinated response. However, if details about the outbreak are unclear, it brings about distrust and uncertainly. In Sinoe county, the cause of the cluster of deaths was not immediately established. It was reported that the health problem in Sinoe was a probable meningitis but outside of the case definition of meningitis. Later, it was indicated that the cluster of deaths was attributed to meningococcal septicemia, thus creating doubts and misinformation in the minds of people. The risk communication response was negatively affected because the evidence for the source of the health problem was unclear. However, in Grand Kru, the response was timely because it was confirmed from the outset that the disease was meningitis. The health promotion team responded timely by disseminating meningitis messages in less than 24 hours. This swift response helped community members to take actions in protecting themselves and their families which resulted in the prompt interruption of the outbreak. Additionally, because both of the health promotion focal persons in the two affected counties were trained in risk communication, they were in a better position to draw up a plan and work with the community to prevent the disease and interrupt the transmission. Partners’ support also helped to quickly respond to the outbreak in a timely manner. Experience from the two outbreaks underscore how critical it is that risk communication plans incorporate the different phases of a response to ensure adequate preparedness and response. These include before the outbreak based on forecasts, during the alert phase leading to the outbreak to enhance control, and after the outbreak to ensure a clear exit strategy and promoting community resilience.

## Conclusion

Risk communication and health promotion strategies form an integral part of any public health response. They provide life-saving information to people in affected communities for proactive actions to protect themselves. Lessons learned from the Ebola and post-Ebola responses clearly showed that health promotion and risk communication strategies were useful in developing and disseminating key messages, engaging communities, and managing rumors so that people can take informed decisions to mitigate the effects of public health threats. Going forward, it is important to incorporate culturally-appropriate health promotion and risk communication strategies in our preparedness and response efforts during emergencies and non-emergencies. This will provide the basis for an effective communication to the public at all times for better outcomes.

### What is known about this topic

- Liberia introduced and implemented successfully the risk communication strategies during the Ebola virus disease outbreak.

### What this study adds

- How the counties in are using the risk communication strategies after the Ebola virus diseases outbreak;
- Best practices on implementation of risk communication strategies in Liberia after the Ebola virus diseases outbreak;
- Challenges faced by the country to implement the risk communication strategies after the Ebola virus disease outbreak.

## Competing interests

The authors declare no competing interest.
